# Seasonal variation and risks of potentially toxic elements in agricultural lowlands of central Cameroon

**DOI:** 10.1007/s10653-022-01473-9

**Published:** 2023-01-12

**Authors:** Annie Stephanie Nana, Timo Falkenberg, Andrea Rechenburg, Joshua Ntajal, Juliet Wanjiku Kamau, Anne Ayo, Christian Borgemeister

**Affiliations:** 1 Center for Development Research (ZEF), University of Bonn, 53113 Bonn, Germany; 2 Laboratory of Biotechnology and Environment, Department of Plant Biology, Faculty of Science, University of Yaoundé I, 812 Yaoundé, Cameroon; 3 Institute of Hygiene and Public Health, Medical Faculty, University of Bonn, 53127 Bonn, Germany; 4 Department of Geography, University of Bonn, 53115 Bonn, Germany

**Keywords:** Agricultural soils, Potentially toxic elements, Pollution index, Ecological risk, Lowlands, Cameroon

## Abstract

Intensive urban agriculture is expanding in Cameroon, which increases the risks of environmental pollution, particularly in the lowlands. This study assessed the seasonal characteristics of some potentially toxic elements and their ecological risks during the dry and wet seasons in urban agricultural soils in four lowlands in Yaoundé. The area and seasonal variation of some potentially toxic elements (Cu, Pb, Zn, Cd, Cr, and Ni) were evaluated using mixed-effects modeling. The pollution status of these elements in the soil was evaluated and interpreted using parameters such as the single pollution (SPI) and Nemerow composite pollution (NCPI) indices. In addition, the effect of these metals in the environment was assessed using the potential ecological risk index (PERI). The distribution of all the studied elements showed an area effect, while a seasonal effect was only noticeable for Cu. The concentration of Cu, Pb, and Zn was higher than the recommended threshold values in one of the areas sampled, while Cr exceeded these threshold values in all areas during both seasons. Cr exhibited the highest SPI among all studied elements during the dry and wet seasons in all areas. Except for the NCPI in one area, where grade V (strong) soil pollution was recorded during the wet season, the soil from all the study areas exhibited a slight pollution level (grade III) with a mean NCPI between 1 and 2. The PERI value of all areas was below the reference values during both seasons, suggesting an overall low potential contamination risk of the soil biota. The results indicate that preventive action to limit the heavy metal contamination in the lowlands of Yaoundé needs to be taken, for instance, through the promotion of more sustainable use of agrochemicals like pesticides and mineral fertilizers.

## Introduction

Some potentially toxic elements (PTEs) are ubiquitous in the environment. They originate both from natural and anthropogenic activities, and the latter are the primary sources of contamination of agricultural soils (Chheang et al., 2021; Liu et al., 2021). These activities include emissions from diverse sources such as industrial activities (mining, smelting, foundries) and agricultural activities, including the application of fertilizers, pesticides, biosolids, manure, and wastewater (Gupta et al., 2019; Sharma et al., 2017; Tóth et al., 2016; Zheng et al., 2020). Heavy metals are potentially toxic pollutants that are persistent in the environment, non-degradable and can bioaccumulate (Ali et al., 2019; Chandel et al., 2021). They can be classified as essential and non-essentials based on their roles in biological systems (Ali et al., 2019). Most of them, i.e., arsenic (As), cadmium (Cd), chromium (Cr), copper (Cu), mercury (Hg), nickel (Ni), lead (Pb), and zinc (Zn), are listed among priority pollutants to control by the United States Environmental Protection Agency (USEPA, 2014). Nevertheless, Ni, Cu, and Zn are essential micronutrients or trace elements for plants, while Cd, Pb, Cr, and As have no known biological roles (Alotaibi et al., 2021). Since they can be toxic, cannot be degraded, and persist in the environment, they either accumulate in biota, soil or leach into the groundwater, causing a combination of environmental and human health problems (Chandel et al., 2021; Islam et al., 2018).

Potentially toxic elements contamination in agricultural soils has become a significant concern worldwide. In developing countries, there is often little awareness and knowledge about these elements and their consequences for the environment and human health (Ali et al., 2019; Eqani et al., 2016). Growing food in PTEs contaminated media leads to bioaccumulation of these elements in the food chains. In Yaoundé, the capital of Cameroon, lowland areas in the valleys constitute the main urban agricultural production zones. They are also often the receptacle of various municipal waste due to the absence of waste treatment technologies. Additionally, farmers in these areas often misuse fertilizers and pesticides during crop production (Nana et al., 2022), resulting in PTEs accumulation in the soil. Various tools, including the pollution indices (Hu et al., 2017) and ecological risk indices (Hakanson, 1980), have been used in soil monitoring to assess the environmental risk of these elements. In a previous study of Yaoundé's lowlands, moderate levels of some PTEs contamination were found (Aboubakar et al., 2021a). Similarly, various extents of pollution have been reported in agricultural soils from other Cameroonian cities (Asongwe & Yerima, 2016; Noubissié et al., 2016). Yet little is known on the seasonality of PTEs contamination in agricultural lowland soils in Cameroon, especially those in and around Yaoundé. Therefore, this study aimed to assess the concentrations of six common elements (Pb, Cd, Cu, Zn, Cr, and Ni) in cultivated lowland soils of Yaoundé and their potential ecological risk during the dry and wet seasons.

## Materials and methods

### Study areas

**Fig. 1:**
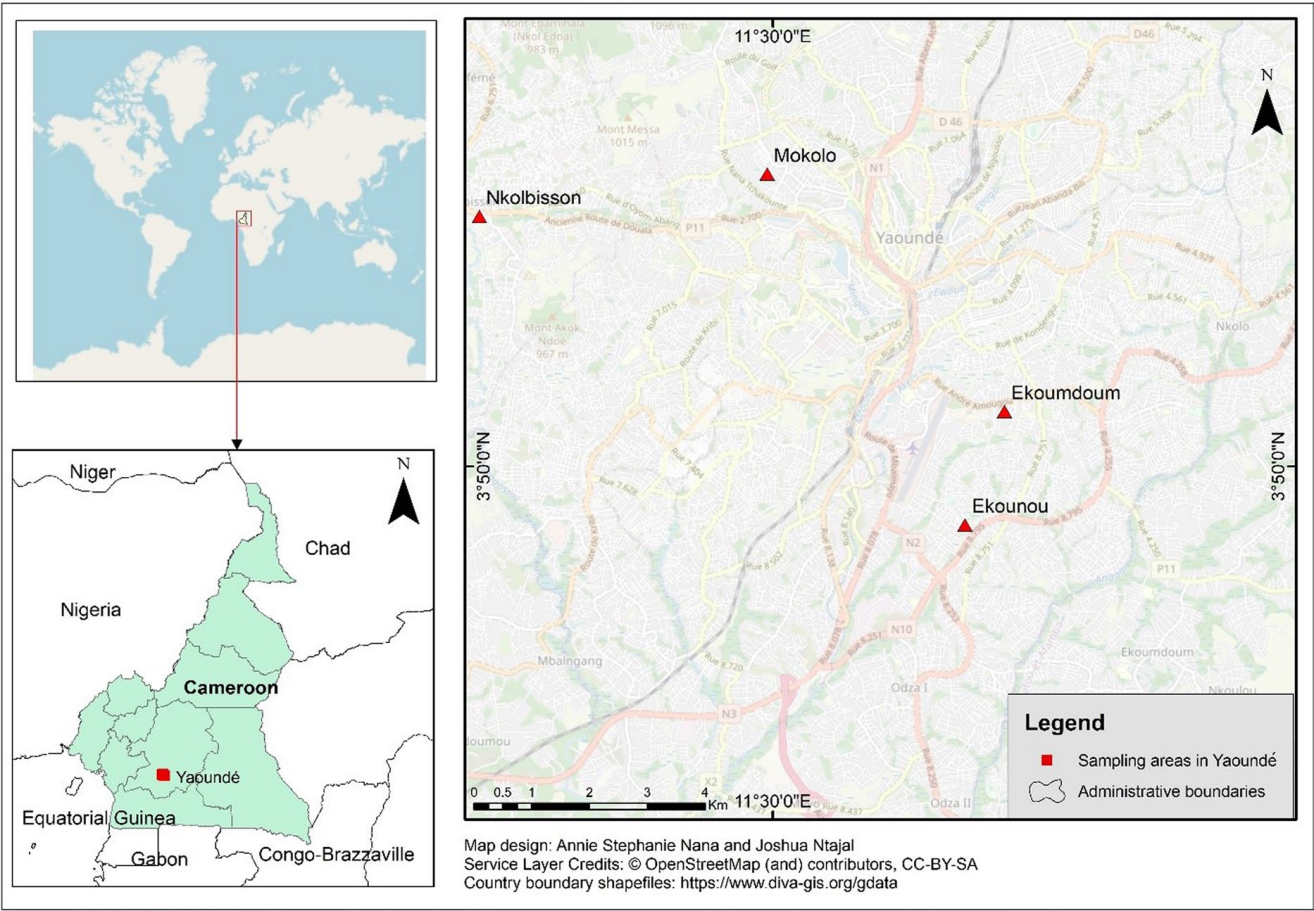
Map of the study area showing the sampling sites

This study was conducted in Yaoundé, the chief town of the Mfoundi division, located in the central region of Cameroon. The city covers about 256 km^2^ pinpointed between latitudes 4°45´N and 4°00´N and longitudes 11°20´E and 11°40´E with an elevation of about 760 m above sea level (m.a.s.l.) (Fig. 1). The city has an equatorial climate with a classical bimodal structure comprised of two alternating dry and wet seasons. The long dry season occurs from December to February and the short dry season from July to August. The two rainfall regimes extend from September to November and from March to June. The full length of these seasons varies, among others, due to global warming. The mean annual rainfall is about 1564.7 mm, with an average temperature of 23.5 °C (Abossolo et al., 2015). The soil in Yaoundé's lowlands is hydromorphic with a mixture of fine sand and organic material in decomposition, while the drylands are primarily ferralitic (Mfopou et al., 2017). The dense hydrologic network in and around Yaoundé is comprised mainly of the Mfoundi river and its perennial tributaries. The four study areas are located in the districts of Yaoundé II (Mokolo), IV (Ekounou and Ekoumdoum), and VII (Nkolbisson) of the Mfoundi division.

These areas were chosen based on several inclusion criteria, such as continuous agricultural production during both the dry and wet seasons (Nana et al., 2022) (Table 1).

**Table 1:** Characteristics of the studied lowland areas in Yaoundé

Variables	Lowland areas			
	Mokolo	Nkolbisson	Ekounou	Ekoumdoum
Location	Yaoundé II	Yaoundé VII	Yaoundé IV	Yaoundé IV
GPS coordinates Longitude (N) Latitude (E) Altitude (m)	11° 49´ 3° 88´ 738	11° 45´ 3° 87´ 696	11° 53´ 3° 83´ 691	11°54´ 3° 84´ 700
Distance from the city center (km)	2–3	7–8	4–5	8–9
Urban settings	Informal settlements	Informal settlements	Peri-urban interfaces	Peri-urban interfaces
Population-level	High	Medium	High	Low
Area size (ha)	1	2	4	6
Farming activity	Low	Medium	High	High
Irrigation water	Groundwater	Surface and groundwater	Surface and groundwater	Surface and groundwater
Level of fertilizer and pesticide application	High	Medium	Low	High
Pollution sources	Craft activities: tannery, waste; oil from open cars repairs, wheels burned, and irons incinerated	Discharge of solid waste and raw sewage; leachate from the dumpsite	Waste dumpsite, road, and surrounding sanitation	Road and surrounding households' sanitation

### Sampling and analysis

From July to August (short dry season) and September to October (short wet season) of 2019, 60 soil samples were randomly collected twice at the beginning of each short season. The collection methods were adapted from the ISO 18400–202 standard (ISO 18400–202, 2018) and the techniques developed by (Aynekulu et al., 2011, 2016). These methods were adapted to the soil conditions of the selected lowland areas and the farmers' availability and consent to participate.

The sampling depth varied between 0 and 20 cm layer for arable soil as recommended for the soil investigation depths in agricultural use (Aynekulu et al., 2016). Samples were collected twice in four months (July and September), with an auger in each of the 60 sampling points distributed across the four areas, i.e., 10 in Mokolo, 12 in Nkolbisson, 16 in Ekounou, and 22 in Ekoumdoum. Four sub-samples weighted approximately 125 g were well mixed to obtain a bulk of composite samples of 500 g, which were packaged into transparent zip-lock plastic bags. The composite sampling of topsoil reduced the number of samples and consequently the analytical costs. Four cores (sub-samples) were collected from the center point, A. In the end, the "composite sample A" corresponded to a mixture of soil from the four sub-plots (A = a_1_ + a_2_ + a_3_ + a_4_) associated with core A. The packaged samples were then transported to the Yaoundé laboratory of the International Institute of Tropical Agriculture (IITA) in a cooler box for pretreatment and analysis.

#### Sample preparation

The pretreatment was conducted following the steps described in ISO 1146 standard (ISO 11464, 2006). Composite soil samples were air-dried at room temperature (25–30 °C) for two weeks in the laboratory and sieved through a 2-mm sieve. Further, they were finely crushed with a rolling pin through a 0.25-mm mesh. Then, 500 mg of < 0.25 mm soil sample were packed and preserved for future analysis.

#### Sample analysis

The conventional aqua regia digestion methods (Chen & Ma, 2001) were performed in 250-ml glass beakers. Soil samples were digested in an aqueous acidic solution to determine the heavy metal contents. Five hundred (500) mg of soil sample was digested in 12 ml of aqua regia on a hotplate at 110 °C under a fume hood for three hours. After evaporation to near dryness, the sample was soaked with 20 ml of 2% nitric acid to convert metal ions into their highly soluble nitrate salts. After filtration through a Whatman n^o^ 0.42 paper, the sample was diluted with deionized distilled water (< 0.5 µS/cm) in a 100-ml volumetric flask. After calibrating the digest samples, total Cd, Cr, Cu, Ni, Pb, and Zn were analyzed inductively via coupled plasma-optical emission spectroscopy (ICP-OES; PerkinElmer Optima 8000) according to the certified standards.

Quality control included soaking glassware and plastic containers in 5% HNO_3_ and drying these before use for ICP-OES. Deionized water was used in all analyses, and all reagents were of analytical grade. The analytical reagent blanks and soil reference materials were prepared and inserted in each batch of samples for analysis to monitor the accuracy and precision of the analytical methods used. Reference soil materials, namely ISE 993 sandy soil from WEPAL (Wageningen Evaluating Programs for Analytical Laboratories), included four external reference samples and one certified sample from the International Soil Exchange Program. Triplicate analysis was performed. All the percentage of recovery obtained for these reference materials was about 75%.

All results obtained were expressed in milligrams per kilogram (mg/Kg) at the respective wavelengths mentioned on the result sheet. The detection limit for Cu, Cd, Zn, Cr, Ni and Pb was 0.26, 0.07, 2.13, 0.32, 0.27 and 1.15 mg/kg, respectively.

### Data analysis

#### Geospatial analysis of heavy metal distribution in the soil

Mapping the spatial distribution of heavy metal concentration in soils in a given area is an important process toward understanding the spatial variability and levels of chemical contamination to inform decision-making. The ordinary kriging method was adopted for geostatistical analysis of the element’s distribution in soils, using the spatial analyst tool in ArcGIS (10.8). Ordinary kriging is used to predict values for unsampled locations. The ordinary kriging is frequently used due to its robustness, unbiased predictions, and capability of smoothening out local variations in the field. It was employed with the normal distribution semi-variogram model embedded in ArcGIS. The model outputs were classified into ten classes using equal intervals. The same methodological steps were repeated to obtain the distribution of PTE concentrations (mg/kg) in the soils for Yaoundé's dry and wet seasons.

#### Pollution assessment

The degree of the PTEs contamination in the soils was estimated using two pollution indices, i.e., the SPI and the NCPI.

The SPI assessed the degree of these elements’ contamination in soils for a single pollutant. It is the ratio of an element's concentration in a soil sample to the standard (background) value of the corresponding element (Hu et al., 2017). It allows identifying which heavy metal represents the highest threat to a soil environment (Kafle et al., 2022) (Table 2).

**Table 2:** Classification of the single pollution index (SPI) and the Nemerow composite pollution index (NCPI)

SPI value	Grade	Class	NCPI value	Grade
SPI ≤ 1	Clean	I	NCPI ≤ 0.7	Safe
1 < SPI ≤ 2	Slight pollution	II	0.7 < NCPI ≤ 1	Warning line
2 < SPI ≤ 3	Moderate pollution	III	1 < NCPI ≤ 2	Slight pollution
SPI > 3	Strong pollution	IV	2 < NCPI ≤ 3	Moderate pollution
		V	NCPI > 3	Strong pollution

The SPI was calculated using Eq. (1):

1 $$ {\text{SPI}} = \frac{{{\text{CS}}i}}{{{\text{SS}}i}} $$

where SPI is the soil pollution index, CS*i* (mg/kg) is the measured concentration of element *i*, and SS*i* (mg/kg) is the standard value of element *i* according to the Finnish Ministry of the Environment (MEF, 2007). Following Tóth et al., (2016: p 300), these thresholds (Table 4) were chosen within the various options (standards) as a suitable standard for heavy metal concentrations in agricultural soils with implications for food safety.

The NCPI or integrated pollution index is a comprehensive index used to classify the soil according to the PTEs contamination (Hu et al., 2017). It highlights the importance of the most contaminated elements (SPImax) while considering all the individual evaluation factors (Zhang et al., 2019). It was calculated using Eq. (2).

2 $$ {\text{NCPI}} = \sqrt {\frac{{\left( {{\text{SPImax}}} \right)^{2} + \left( {{\text{SPIm}}} \right)^{2} }}{2}} $$

where *S*PImax is the maximum SPI value and SPIm is the mean SPI of all the studied potentially toxic elements(Dey et al., 2021; Li et al., 2018). SPI and NCPI classifications are defined in Table 2.

#### Potential ecological risk

The potential ecological risk index (PERI) was used to assess the risk of the PTEs in the study areas' soils (Hakanson, 1980). It evaluates the level of the elements pollution by considering their toxicity, synergy, and ecological sensitivity. PERI is the sum of each PTE’s ecological risk factor index (E_r_) (Aboubakar et al., 2021a; Liu et al., 2021; Weissmannová et al., 2019). E_r_ and PERI were calculated using Eqs. (3) and (4), respectively.

3 $$ E_{r}^{i} = T_{r}^{i} \times {\text{SPI}} $$

4 $$ {\text{PERI}} = \mathop \sum \limits_{i = 1}^{n} E_{r}^{i} $$

where $$ E_{r}^{i}$$ represents the single ecological risk index; $$T_{r }^{i}$$ is the toxic response factor defined as 5, 5, 5, 2, and 1 for Cu, Pb Ni, Cr and Zn, respectively (Liu et al., 2021); and SPI is the single pollution index calculated as described in Eq. (1). $$E_{r}^{i}$$ and PERI can be classified as defined in Table 3.

**Table 3:** Classification of single and potential ecological risk index

Risk level	Low	Moderate	High	Very high	Disastrous
Grade	I	II	III	IV	V
Er	< 30	30–60	60–120	120–240	> 240
PERI	< 100	100–200	200–400	> 400	

#### Statistical analyses

All data were analyzed using Stata version 15.1 (StataCorp LLC, 2017). The effect of seasons and areas on the distribution of the PTEs was tested and estimated using a robust in case of heteroscedasticity two factorial mixed-effect models with interaction (Rothman, 2012). This model was chosen because season and division were fixed factors, while farmers were random factors. Model fit was determined using an alpha value of 0.05, with a p-value < 0.05 indicating the level of statistical significance. The complexity in the model was reduced by eliminating less relevant inputs and avoiding overfitting (Rothman, 2012). To correct the normality, the models were bootstrapped (Davis et al., 2010). A pairwise comparison of marginal effects was then used to assess the difference between seasons and areas at a 95% confidence interval.

## Results and discussion

### Potentially toxic elements in the study areas

**Fig. 2:**
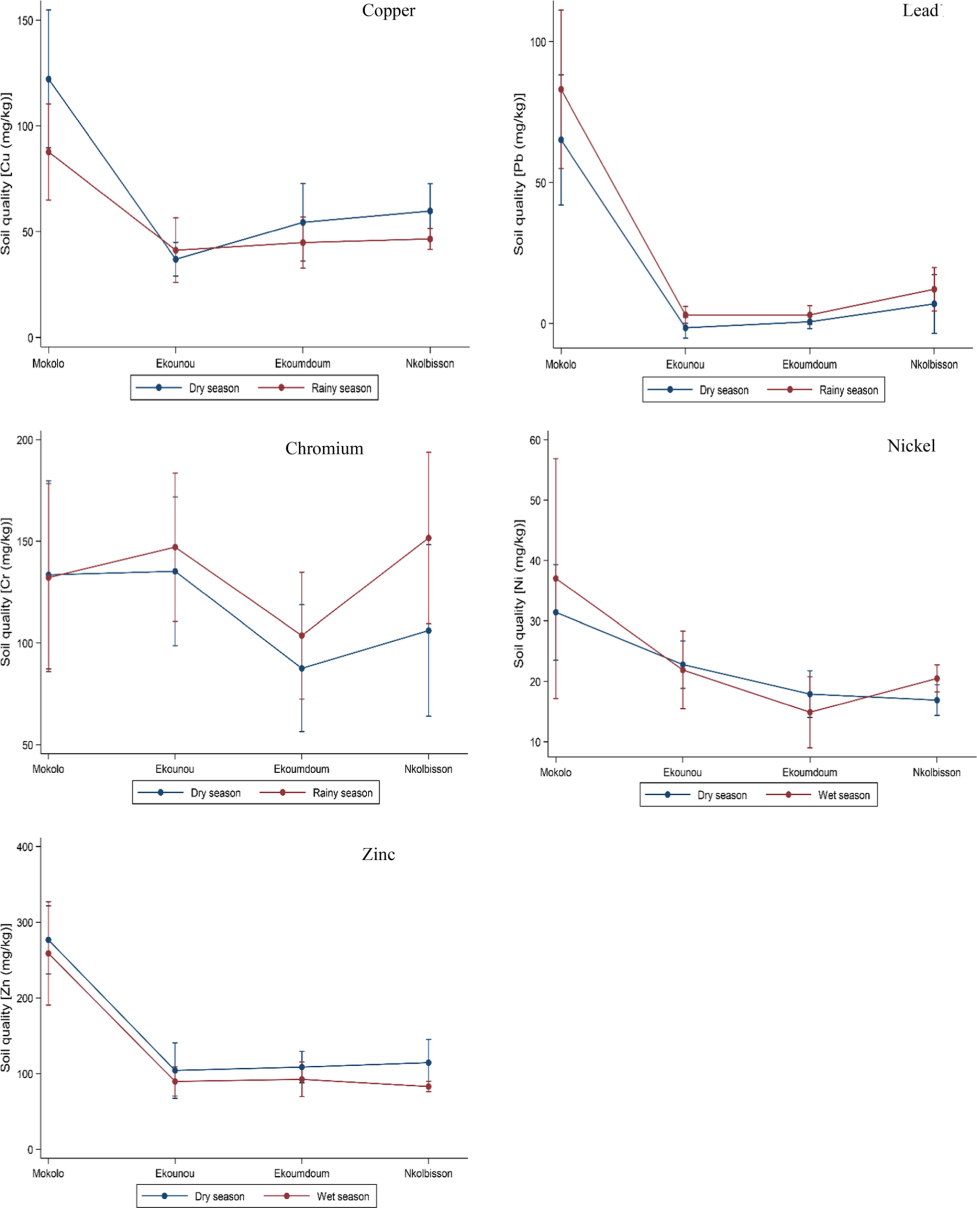
Margin plots showing the seasonal variation of the studied elements in the agricultural soil in the four areas of Yaoundé (mean value with 95% confidence intervals)

The concentrations of the five studied PTEs in the agricultural soil samples from the four Yaoundé lowland areas are presented in Fig. 2. The highest concentrations of Cu, Pb, Zn were recorded in dry season soil samples from Mokolo, possibly because of the significantly higher fertilizer and pesticide use there compared to the other three studies areas (Nana et al., 2022). The Food and Agriculture Organization (FAO) reported that 50% of most commonly agricultural soils are Zn deficient. Thus, farmers rely upon Zn-fertilizer to enhance the plant's growth and development (IZA, 2015). Moreover, Cu is an essential oligo element that has been used in agriculture as fungicides and bactericides to protect plants (La Torre et al., 2018). Furthermore, a recent study demonstrated that fertilizer (nitrogen, potash, and organic) contributes to adding Pb in agricultural soils (Chen et al., 2021).

Run-off might affect the PTEs removal capacity from farmlands, leading to higher concentrations of these elements during the dry season. Evaporation is also more intense during the dry season, leading to more concentrated soil solutions. Meanwhile, rainfall might dilute the soil solution and contribute to the leaching of the PTEs from the soil into the groundwater.

The highest concentrations of Cr and Ni were recorded during the wet season in Nkolbisson and Mokolo soils, respectively, suggesting that these elements are highly integrated into the sediments. In South-West Nigeria, Oluyemi et al. (2008) similarly found high concentrations of Cr during the wet season. In agricultural soils, Cr and Ni generally originate from the parent material and the application of agrochemicals, especially pesticides. Defarge et al. (2018) reported residues of Cr (up to 40 times), Ni (up to 62 times), and Pb (up to 11 times) above the permitted level in some glyphosate-based herbicides. These agrochemicals and other waste residues leaching from the surrounding residential areas and construction sites situated in higher altitudes add these elements to the agricultural soils, and their presence can be found in the environment decades later (Dos Reis et al., 2017).

The total concentration of the PTEs varied among the study areas in the order of Mokolo > Nkolbisson > Ekounou > Ekoumdoum. Similarly, the mean concentrations of these elements in these soils were in the order of Zn > Cr > Cu > Ni > Pb in the dry and Cr > Zn > Cu > Pb > Ni in the wet season.

High variability in terms of coefficient of variation (CV) was observed within samples from the same area and between samples of different areas. Based on the CV values, the soil sample can be classified as having low (CV ≤ 25%), moderate (25% ˂ CV ≤ 75%), or high (CV > 75%) variability (Mamut et al., 2018). In this study, the CV values ranged from 35 to 85%, with moderate variability for Zn (35), Cr (42), Cu (43), and Ni (46) and high variability for Pb (85). Low CV values of these PTEs in soil indicate that natural sources are dominant, whereas high CV values point to anthropogenic sources that affect the distribution of these elements (Baltas et al., 2020). Thus, the moderate to high variability observed in this study suggests a strong influence of anthropogenic activities, including the use of untreated water for irrigation, pesticide and fertilizers usage, and the informal industries in the surrounding areas.

The concentrations observed in the agricultural soils of the Yaoundé lowlands were compared with the permissible limits set by the Finnish Ministry of Environment (MEF, 2007). The concentration of Cr was higher, and Ni was lower than the permissible limits in all the areas during both dry and wet seasons. With 152 mg/kg, the highest concentration of Cr was observed at Nkolbisson. Although in their study on farming practices and disease prevalence in Yaoundé, Nana et al. (2022) reported a high percentage of farmers use fertilizer in Nkolbisson, this area was also irrigated by river water which potentially was contaminated by domestic wastewater and sewage sludge from the nearby residential areas. Moreover, most farms in Nkolbisson belong to the neighboring Cameroonian Institute of Agricultural Research for Development and are frequently used as experimental fields, which possibly explains the significantly higher concentration of Cr compared to the other studied lowland areas. The concentrations of Cr, Ni, Zn, and Cu in Nkolbisson were higher than those reported by Aboubakar et al. (2021b) in the same area (cf. Table 4). Yet, the same authors observed Cd and Pb in higher concentrations than the concentrations obtained in this study (except for Mokolo). They noted that Cd in the soil varied between 0.02 and 0.25 mg/kg, whereas it remained below the detection limits in this study. This difference between our results and those of Aboubakar et al. (2021b) might be explained by the relative balance between processes such as sorption, leaching, and plant uptake, which are also strongly affected by other soil properties as well as the type of agrochemicals used. For instance, Kubier et al. (2019) reported that elevated Cd contents in soil are generally linked to elevated pH and abundance of carbonates, organic matter, and clay minerals, and Tóth et al. (2016) demonstrated that significantly more Cd in agricultural soils is caused by the different origins of phosphate fertilizers used in agriculture.

**Table 4:** Average concentration of the studied elements in mg/kg in the agricultural lowland soils compared to other recent studies

Country/city	Areas	Seasons	Cu	Pb	Ni	Cr	Zn	Cd	Authors
Wavelengths	–	–	327.39	220.35	231.60	267.72	213.86	228.85	
Reference materials (ISE 993 sandy soil)			64.04	62.08	31.44	113.95	167.84	10.65	
Cameroon/Yaoundé	Mokolo	Dry	122.2 (40.8)	65.1 (28.9)	31.4 (9.9)	133.5 (21.6)	276.6 (56.4)	nd	Current study
		Wet	87.7 (28.5)	83.1(35.2)	37.0 (24.9)	132.1(42.5)	258.9 (85.5)	nd	
	Ekounou	Dry	36.9 (12.1)	1.4 (3.7)	22.8 (5.9)	135.2 (46.6)	104.1 (55.5)	nd	
		Wet	41.2 (23.2)	3.6 (3,8)	21.9 (9.7)	147.1 (48.0)	89.6 (29.1)	nd	
	Ekoumdoum	Dry	54.4 (31.9)	2.0 (3.0)	17.9 (6.8)	87.6 (48.6)	108.6 (36.2)	nd	
		Wet	44.8 (21.2)	4.0 (4.7)	15.2 (9.7)	103.6 (76.5)	92.4 (39.7)	nd	
	Nkolbisson	Dry	59.7 (17.4)	8.8 (12.3)	16.9 (3.4)	106.1 (34.5)	114.4 (47.0)	nd	
		Wet	46.5 (6.4)	12.5 (9.8)	20.5 (3.0)	151.6 (43.2)	82.9(9.3)		
Cameroon/Yaounde	Kolomdom	Dry	5.7 (0.2)	10.9 (2.9)	5.9 (0.2)	2.4(0.6)	27.5(1.6)	0.04(0.01)	Aboubakar et al., (2021b)
	Nkolbisson	Dry	6.7 (0.7)	16.5 (3.4)	11.9 (1.3)	16.5(3.5)	27.6(1.1)	0.07(0.01)	
Cameroon/Bamenda	–	–	–	30.6 (2.0)	–	35.7 (0.5)	–	0.5 (0.5)	Asongwe and Yerima, (2016)
Cameroon/Ngaoundere	Bali	–	10.3 (0.3)	17.5 (0.1)	24.6 (0.0)	–	30.6(2.06)	nd	Noubissié et al., (2016)
	Sabongari	–	19.7 (1.2)	53.4 (6.0)	26.3 (1.3)	–	164.7(8.7)	0.1 (0.0)	
Nigeria/ Ife	Obafemi Awolowo	Dry	875 (0.0)	317.5 (0.0)	128.1 (0.0)	107.5 (0.0)	366.2 (0.0)	11.3 (65.0)	Oluyemi et al., (2008)
		Wet	844 (0.0)	304.5 (0.07)	117.6 (0.0)	181.3 (0.0)	206.6 (0.0)	10.4 (0.0)	
Kenya, Homabay	Homahills	–	12.0–12.9	9.9–10.8	–	9.7–10.1	0.2–46.9	0.5–0.7	Akenga et al., (2020)
Ghana/Tarwa	–		5.1 (1.9)	4.8 (3.5)	2.2 (1.0)	16 (10)	37 (24)	0.03 (0.00)	Bortey-Sam et al., (2015)
Ghana/Accra	Korle Lagoon		3.5 (0.05)	1.3 (0.02)	0.9 (0.05)	3.0 (0.1)	0.83 (0.05)	3.6 (0.58)	Fosu-Mensah et al., (2017)
Threshold values			100	60	50	100	200	10	MEF, (2007)

Values in bracket represent standard deviation**;** – for not applicable; nd for not detected

Cu, Pb, and Zn concentrations were higher than the MEF threshold values in Mokolo but lower in the other lowland areas. Informal waste disposal can potentially explain these high concentrations since the Mokolo site was among the most polluted areas studied, having many informal industries such as tanneries, casting lead and lead products manufacturing, and waste burning activities. These uncontrolled activities might release a considerable amount of Cu, Zn, Ni, and Pb into the environment, thereby contaminating the surrounding agricultural soils (Luo et al., 2012). Contrary to the other studied lowland areas, farmers in Mokolo also reported using only groundwater obtained from shallow dug wells built near the plots for irrigation. Thus, the observed high potentially toxic element contamination might be related to the low groundwater quality contaminated with organic matter and other pollutants drained by wind and run-off during rain.

Moreover, the high levels of Zn, Cu, Pb in Mokolo could have also emanated from agrochemicals and domestic inputs, building materials, and nearby road traffic activities. Farmers in Mokolo reported high fertilizer use on their land (Nana et al., 2022). Qaswar et al. (2020) reported a significant increase in Cu, Zn, Cr, and Cd in soils with the combined application of manure and inorganic fertilizers. The same authors demonstrated that high Pb concentrations are more common among farmers using only inorganic fertilizers than those using mixtures of organic and inorganic fertilizers. Except for Cd, which was below the detection range, the concentrations of Cu, Zn, Ni, Cr, and Pb (the latter only in Mokolo) in this study were higher than those reported by Aboubakar et al. (2021b) in two agricultural soils of Yaoundé (cf. Table 4). Apart from Oluyemi et al. (2008) study in Nigeria, the potentially toxic elements concentrations reported here were generally higher than those reported from other African cities (Table 4). The results of the linear mixed-effect models are reported in Table 5. Although the interaction between areas and seasons was not significant, generally significant area differences in the concentrations of Cu, Cr, Ni, Pb, and Zn were found. However, only the Cu concentrations showed significant seasonal differences. This result could be due to the high amount of rainfall that resulted in the leaching of Cu, which led farmers to increase the application of agrochemicals because it was washed out, resulting in a further increase in the soil. In addition, it could also be explained by soil properties such as pH, texture, and clay that have been reported to affect the retention of Cu in the agricultural soils (Król et al., 2020)**.** In summary, there are clear area effects for all studied potentially toxic elements, but a seasonal effect only for Cu. (More detailed results for specific areas and seasonal differences are reported in Appendix.)

**Table 5:** Contrasts of marginal linear predictions

Variables	Cu			Cr		Ni		Pb		Zn	
	*df*	*F*	*P* > *F*	*F*	*P* > *F*	*F*	*P* > *F*	*F*	*P* > *F*	*F*	*P* > *F*
Areas	3	39.8	**0.000**	8.0	**0.046**	13.3	**0.004**	118.6	**0.000**	89.6	**0.000**
Season	1	4.1	**0.042**	2.6	0.110	0.2	0.646	1.2	0.266	2.2	0.138
Areas # season	3	5.2	0.161	1.9	0.587	5.6	0.130	0.6	0.901	0.8	0.846

Number in bold: statistically significantly different at *p* < 0.05 and *P* < 0.01

### Spatial distribution of some potentially toxic elements in Yaoundé

**Fig. 3:**
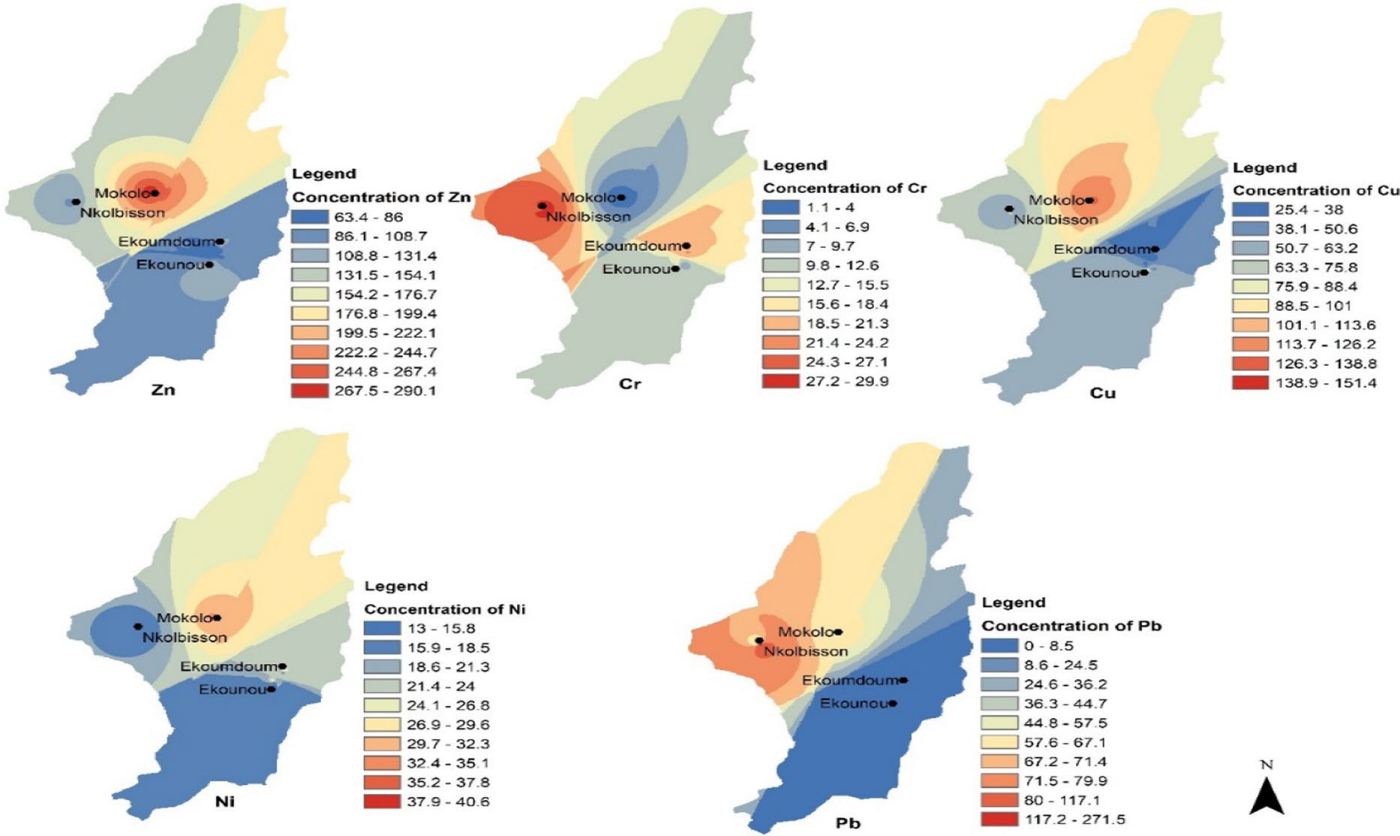
Spatial distributions of the element concentrations (mg/kg) in the soil during the dry season in Yaoundé. Zn = zinc, Cr = chromium, Cu = copper, Ni = nickel, and Pb = lead

**Fig. 4:**
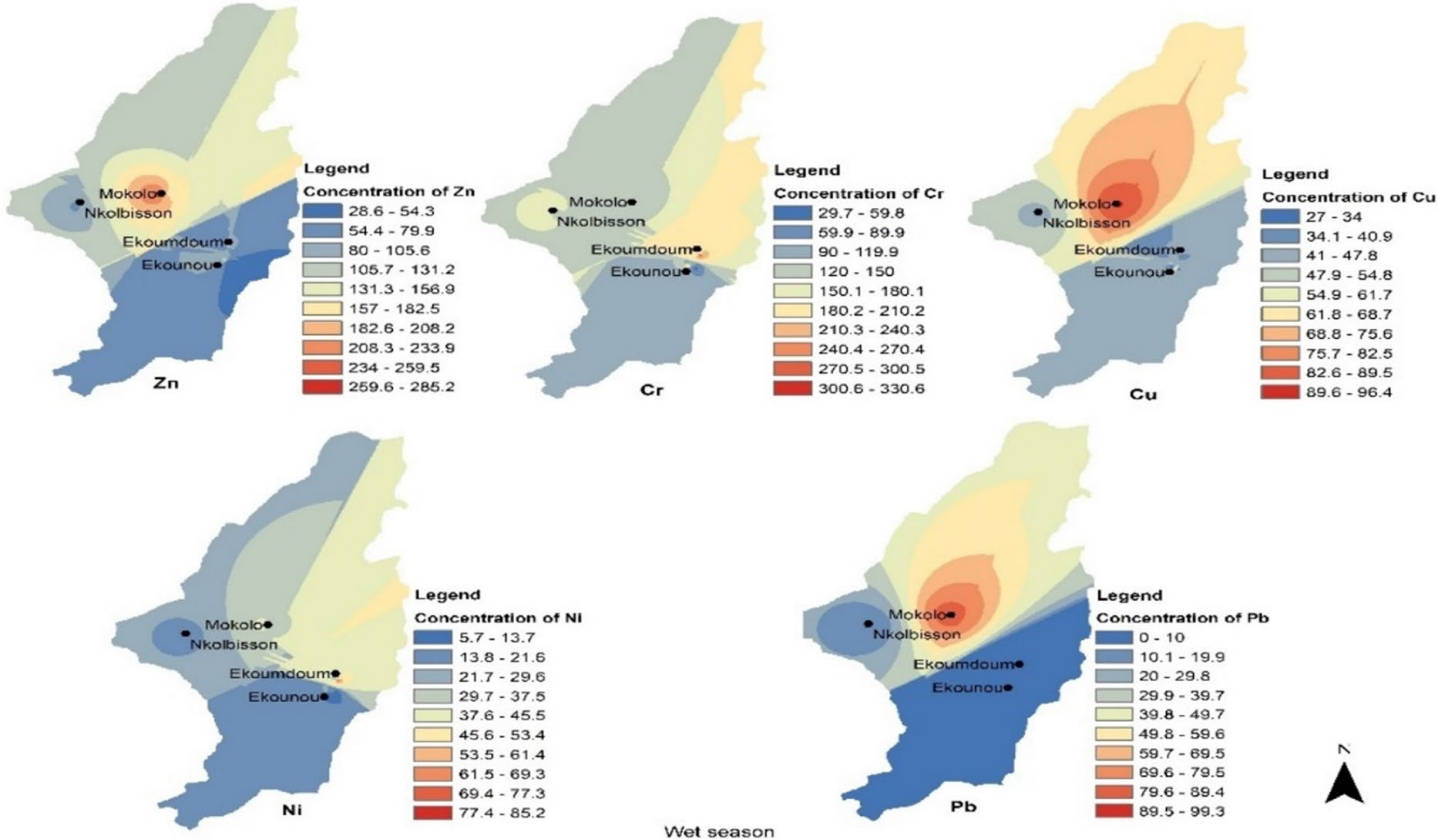
Spatial distributions of the element concentrations (mg/kg) in the soil during the wet season in Yaoundé. Zn = zinc, Cr = chromium, Cu = copper, Ni = nickel, and Pb = lead

Spatial distributions of the studied elements during the dry and wet seasons obtained by the ordinary kriging interpolation analysis are shown in Figs. 3, 4, respectively. These maps visualize the potential distribution of potentially toxic elements in Yaoundé's soils in the Mokolo, Nkolbisson, Ekoumdoum, and Ekounou areas.

Zn and Cu distributions were similar during both dry and wet seasons, with higher concentrations in the central part of Yaoundé (around Mokolo). The similitude recorded in the distribution of Cu and Zn during the dry and wet seasons in this study suggests similar sources of contamination during both seasons and corroborated those reported by Sun et al. (2019) in China. The authors reported that the distribution of Zn and Cu was significantly influenced by their sources of contamination, mostly anthropogenic activities like agricultural inputs such as irrigation and agrochemicals applications that can lead to enrichment of these elements in soils. Although agriculture activities in the studied areas could be the main sources of these elements, they could also originate from other pollution sources such as e-wastes which are frequently burned near the farms. Moreover, as the areas is located in populated neighborhoods, they could also be sourced by building material such as roofing and plumbing, and vehicle wear (from open car repair) that surround the sites.

Cr, Ni, and Pb distributions differed across the seasons, though with generally slightly higher concentrations during the dry season. The observed seasonal variation might indicate some changes in the organic profile or redox potential and pH conditions (Islam et al., 2018). Moreover, the differing Ni, Cr, and Pb concentrations in soils might be attributed to the pollution sources that varied from one area to another as a result of the local farming practices.

### Soil Pollution Indices

The SPI results of the five studied heavy metals, representing the degree of contamination in the soil environment, are summarized in Table 6. Except for Ekounou, all the studied soils were contaminated with Cr during both seasons, suggesting that these areas can be classified as slightly polluted by Cr (grade II). Similarly, except for Ni, the soil in Mokolo was contaminated with all five PTEs during both seasons. In summary, the studied elements in the soils range from clean (SPI > 1) to slightly polluted (1 < SPI ≤ 2).

**Table 6:** Single pollution (SPI) and Nemerow composite pollution (NCPI) indices of soil's heavy metals

Variables		Seasons	Mokolo	Ekounou	Ekoumdoum	Nkolbisson
Single Pollution Index (SPI)	Zn	Dry	1.4 (0.3)	0.6 (0.2)	0.5 (0.2)	0.6 (0.2)
		Wet	1.3 (0.4)	0.5 (0.2)	0.4 (0.1)	0.4 (0)
	Cr	Dry	1.3 (0.2)	0.7 (0.3)	1.4 (0.4)	1.1 (0.3)
		Wet	1.3 (0.4)	0.7 (0.6)	2.1 (1.7)	1.5 (0.4)
	Cu	Dry	1.2 (0.4)	0.6 (0.3)	0.4 (0.1)	0.6 (0.2)
		Wet	0.9 (0.3)	0.5 (0.2)	0.4 (0.2)	0.5 (0.1)
	Ni	Dry	0.6 (0.2)	0.3 (0.1)	0.4 (0.1)	0.3 (0.1)
		Wet	0.7 (0.5)	0.2 (0.2)	0.8 (1.3)	0.4 (0.1)
	Pb	Dry	1.1 (0.5)	0 (0.1)	0 (0.1)	1.3 (2.8)
		Wet	1.4 (0.6)	0.1 (0.1)	0.1 (0.1)	0.2 (0.2)
NCPI		Dry	1.5	1.0	1.5	1.2
		Wet	1.7	1.2	5.0	1.6

Values in bracket represent standard deviation

According to the NCPI classification in Table 2, except for Ekoumdoum soil during the wet season (grade V or strong pollution), the soil from all studied areas was at the slight pollution level (grade III), with the mean NCPI ranging between 1 and 2.

The NCPI grade V record in Ekoumdoum during the wet season was mainly influenced by the high SPI of the Cr element. During both seasons, Cr had the highest SPI among the studied elements, possibly driven by anthropogenic activities like agrochemicals, tanneries effluents, leather tanning and cement products, and other wastewater and sludge containing chromate manufactured products found in the neighboring areas. Moreover, intensive human intervention like unplanned urbanization and population pressure has led to large amounts of waste that are discharged in the environment without treatment. Of all the wastewater treatment plants (13) that existed before 1990 in Yaoundé, almost all are dysfunctional. Although, since 2010, the Municipality of Yaoundé, has restored three of them (Grand Messa, Cité Verte and Biyem-Assi), they remain poorly constructed (under the inflow capacity) and there is no sewage treatment plant of fecal sludge (GFA, 2018). Furthermore, solid waste management in Yaoundé is assigned to a private company (HYSACAM) and many neighborhoods do not benefit from its services, due to their location in outlying areas. Thus, lowlands in Yaoundé receive mixed wastes from the city due to the inefficacy of the municipal and private waste collection services. Thus, this influx possibly further increases the already high heavy metal concentrations in the agricultural soils. According to Prasad et al. (2021), toxic residues entering the freshwater streams subsequently pollute the soils and negatively impact plant growth and animal health, indirectly damaging terrestrial and aquatic ecosystems. Moreover, long-term exposure creates many harmful consequences and acts as mutagenic, teratogenic, carcinogenic substances (Prasad et al., 2021). Overall, this study's findings indicated that the NCPI varied from 1.0 to 5.0 in all areas' overall seasons, suggesting that these agricultural soils were slightly to strongly polluted. These results were similar to those of Aboubakar et al. (2021a), who reported that the NCPI of soil in three cultivated Yaoundé sites studied varied from 1.26 to 7.79.

### Ecological risk

The single ecological risk (Er) and the integrated potential ecological risk (PERI) of the five studied PTEs are shown in Table 7. It represents the sensitivity of various ecosystems to toxic substances and can identify the potential ecological risks caused by PTEs (Mamut et al., 2018).

**Table 7:** Single ecological and integrated potential ecological risks of the studied elements in soil

Variables		Seasons	Mokolo	Ekounou	Ekoumdoum	Nkolbisson
Single ecological risk index (Er)	Zn	Dry	1.4 (0.3)	0.6 (0.2)	0.5 (0.2)	0.6 (0.2)
		Wet	1.3 (0.4)	0.5 (0.2)	0.4 (0.1)	0.4 (0)
	Cr	Dry	2.7 (0.4)	1.3 (0.6)	2.8 (0.9)	2.1 (0.7)
		Wet	2.6 (0.8)	1.5 (1.1)	4.1 (3.4)	3 (0.9)
	Cu	Dry	6.1 (2)	3.1 (1.7)	1.8 (0.6)	3 (0.9)
		Wet	4.4 (1.4)	2.4 (1.1)	2 (1)	2.3 (0.3)
	Ni	Dry	3.1 (1)	1.7 (0.7)	2.2 (0.6)	1.7 (0.3)
		Wet	3.7 (2.5)	1.1 (0.8)	4.2 (6.5)	2 (0.3)
	Pb	Dry	5.4 (2.4)	0.2 (0.3)	0.1 (0.3)	6.3 (14.2)
		Wet	6.9 (2.9)	0.4 (0.4)	0.3 (0.3)	1 (0.8)
PERI		Dry	18.7 (5.0)	6.9 (2.0)	7.4 (1.6)	13.7 (15.4)
		Wet	18.9 (7.3)	5.9 (2.9)	11.1 (9.8)	8.8 (1.6)

Values in bracket represent standard deviation

The average Er and PERI of the cultivated lowlands' soils of Yaoundé during both dry and wet seasons were below the threshold values of 30 and 100, respectively (cf. Table 3). Although Er was classified as Pb > Cu > Ni > Cr > Zn in both seasons, these results indicate that the studied elements induce a low ecological risk in these areas. Similarly, PERI can be classified as Mokolo > Nkolbisson > Ekoumdoum > Ekounou in the dry, and Mokolo > Ekoumdoum > Nkolbisson > Ekounou in the wet season. Although in both seasons Mokolo exhibited the highest ecological risk, the PERI recorded in all the studied areas suggest an overall low potential contamination of the biological communities. A previous study in Yaoundé reported a low to moderate ecological risk in cultivated land (Aboubakar et al., 2021a). These authors highlighted Cd as being the key factor influencing the PERI as its Er was much higher than the other studied elements (Cd, Cr, Cu, Mn, Ni, Pb and Zn), thus causing higher toxicity. As the presence of Cd was below the detection limit in this study, potential ecological risk could be attributed to the toxicity of Pb, which is the second most harmful studied elements.

In contrast, the average concentrations of the elements recorded in Mokolo and the concentration of Cr in all the studied areas during both seasons were above the reference values (cf. Table 4). Similarly, strong pollution was recorded in Ekoumdoum during the wet season, whereas slight pollution was shown in the other areas. Thus, the ecological risk might vary according to location and farming practices, and some of the high values might be attributed to various contaminant sources such as the natural weathering, leaching of rocks, and various discharges from agricultural and artisanal mining activities surrounding the study areas.

## Conclusion

This research assessed the seasonal variation of some PTEs and their ecological risk in four agricultural lowlands in Yaoundé, Cameroon. All studied metals exhibited an area effect in their distribution, while a seasonal effect was only noticeable for Cu. The variability of these metals was classified as moderate to high, suggesting their anthropogenic sources like fertilization, pesticides, wastewater, and other human activities. Furthermore, although the potential ecological risk shows low potential biological contamination, the soil from all areas was slightly polluted, except that from Ekoumdoum, which was strongly polluted during the wet season. Thus, there is need to educate farmers by providing them with more training on the safe use of agrochemicals inputs to reduce the risk of these potentially toxic element contamination of their soils. Further training is required for city dwellers for a sustainable waste management (liquids and solids) and environmental contamination through urban industrial activities.

Moreover, regular monitoring to raise farmers' awareness of the levels of the PTEs in their agricultural soils is recommended. Regulating and adjusting agronomic measures, planting patterns and land-use types in areas where the potentially toxic elements contents are high, could minimize the human health risk by better controlling the pollution source. Therefore, this study serves as an early warning of PTEs contamination of agricultural soils in the lowlands of Yaoundé. Future studies should monitor the dynamics of pollution and the transferability of these elements from the agricultural soil to the crops, and the potential health risks in humans and livestock.

## Data Availability

Data are available from the authors with the permission of collaborators.

## Appendix: Seasonal and areas difference in the studied element content in the agricultural soil of the lowlands in Yaoundé

**Table:**

Variables		Cu		Pb		Cr		Ni		Zn	
**Areas**	**Season**	Contrast	*P* > *t*	Contrast	*P* > *t*	Contrast	*P* > *t*	Contrast	*P* > *t*	Contrast	*P* > *t*
Mokolo	Dry vs wet	− 34.47	0.118	17.96	0.427	− 1.35	0.967	5.62	0.612	− 17.81	0.712
Ekounou	Dry vs wet	4.26	0.534	2.21	0.070	11.88	0.646	− 0.90	0.839	− 14.50	0.284
Ekoumdoum	Dry vs wet	− 9.592	0.345	2.07	**0.024**	16.07	0.467	− 2.68	0.235	− 16.20	0.208
Nkolbisson	Dry vs wet	− 13.22	0.051	3.64	0.489	4.548	0.132	3.59	**0.022**	− 31.52	0.041
**Season**	**Areas**										
Dry	Ekounou vs Mokolo	− 85.24	**0.000**	− 63.68	**0.000**	1.728	0.953	− 8.62	0.056	− 172.56	**0.000**
Dry	Ekoumdoum vs Mokolo	− 67.78	**0.000**	− 63.12	**0.000**	− 45.9	0.104	− 13.50	**0.003**	− 168.10	**0.000**
Dry	Nkolbisson vs Mokolo	− 62.44	**0.000**	− 56.28	**0.000**	− 27.4	0.384	− 14.50	**0.001**	− 162.22	**0.000**
Wet	Ekounou vs Mokolo	− 46.51	**0.001**	− 79.44	**0.000**	14.95	0.613	− 15.13	0.155	− 169.26	**0.000**
Wet	Ekoumdoum vs Mokolo	− 4.29	**0.001**	− 79.02	**0.000**	− 28.5	0.309	− 21.81	**0.038**	− 166.49	**0.000**
Wet	Nkolbisson vs Mokolo	− 41.19	**0.001**	− 70.61	**0.000**	19.47	0.535	− 16.53	0.105	− 175.93	**0.000**
Dry	Ekoumdoum vs Ekounou	17.46	0.086	0.56	0.716	− 47.6	0.052	− 4.89	0.082	4.47	0.835
Dry	Nkolbisson vs Ekounou	22.8	**0.003**	7.40	0.127	− 29.1	0.300	− 5.88	**0.013**	10.34	0.672
Wet	Ekoumdoum vs Ekounou	3.61	0.717	0.41	0.826	− 43.4	0.075	− 6.67	0.123	2.76	0.856
Wet	Nkolbisson vs Ekounou	5.32	0.516	8.83	0.025	4.516	0.872	− 1.40	0.687	− 6.68	0.522
Dry	Nkolbisson vs Ekoumdoum	5.343	0.64	6.85	0.150	18.56	0.481	− 1.00	0.673	5.88	0.756
Wet	Nkolbisson vs Ekoumdoum	17.1	0.799	8.42	0.034	47.96	0.072	5.28	0.084	− 9.44	0.438

Number in bold: statistically significant difference at *p *< 0.05.
